# Effects of exercise interventions for specific cognitive domains in old adults with mild cognitive impairment: A protocol of subgroup meta-analysis of randomized controlled trials

**DOI:** 10.1097/MD.0000000000013244

**Published:** 2018-11-30

**Authors:** Xiang-Lian Zhou, Li-Na Wang, Jie Wang, Xin-Hua Shen, Xia Zhao

**Affiliations:** a School of Nursing, Medical College of Huzhou University; b Department of Psychosomatic Diseases, Huzhou Third People's Hospital, Zhejiang Province, China.

**Keywords:** aged, cognitive, exercise, meta-analysis, mild cognitive impairment

## Abstract

**Background:**

Mild cognitive impairment (MCI) is the clinical prognosis that leads to dementia. Early intervention of MCI is critical to protect against dementia. Exercise intervention has gained popularity for the management of MCI. Most current studies have focused only on improvements made by exercise interventions on the global/general cognitive function and/or a specific cognitive function. However, no studies have been performed on a subgroup analysis of the effects of exercise interventions on different cognitive domains of the elderly with MCI. The exploration of this issue will help to clarify the influence and through a preliminary analysis identify the level of influence of exercise interventions on particular cognitive domains, and it will provide the theoretical framework for the construction of precise exercise intervention strategies for MCI patients.

**Methods:**

A systematic review of electronic databases (MEDLINE, Embase, The Cochrane Library, Web of Science, CNKI, the Wan Fang Database, and CBM), supplemented by expert contact, reference and citation checking, and gray literature searches have been conducted. There is no restriction on language or publication status. Cochrane Handbook for Systematic Reviews of Interventions and add another 3 items according to PEDpro, including “the type of statistical analyses used (true intention-to-treat vs other),” “eligibility criteria,” and “baseline comparability,” are used to assess the risk of bias. Primary outcomes of interest are standardized measurements of executive functions, memory, language, processing speed, and attention. If possible, we conduct a meta-analysis to synthesize the evidence for each outcome.

**Results:**

This study will provide a high-quality synthesis of current evidence of exercise for MCI patients.

**Conclusion:**

The conclusion of this systematic review will provide evidence to judge whether exercise is an effective intervention for patient with MCI and preliminary ranking of the effects of exercise on specific cognitive domains.

**Prospero registration number:**

CRD42018093902

## 1 Introduction

### 1.1 Description of the condition

The 2016 World Alzheimer Report reported that 47 million people live with dementia worldwide. With the accelerating trend of population ageing, that number is expected to increase to more than 131 million by 2050. Dementia also has a huge economic impact. The total estimated worldwide cost of dementia is US$818 billion, and it will be a trillion-dollar disease by 2018.^[1]^ However, in 2015, a systematic review showed that medication therapy produces small, short-lived improvements in cognitive function in mild to moderate dementia, which is not relevant for clinically meaningful effects.^[2]^

At present, for the prevention and treatment strategy of dementia, the basic research and clinical research have turned to the preclinical stage—the mild cognitive impairment (MCI) domain. MCI is an intermediate stage between normal brain ageing and dementia. It is the prodromal stage of various dementias and neurodegenerative diseases, and this cognitive impairment is more severe than normal ageing. Although it is often accompanied by mild memory impairment, it will gradually develop into severe cognitive impairment and functional impairment in the following years.^[3]^ It is reported that 10% to 15%, 60.5%, and 100% of patients with MCI will develop full dementia within 1 year, 5 years, and 9.5 years, respectively, after initial diagnosis of MCI.^[4]^ Therefore, the cognitive management of MCI is particularly important for the prevention of dementia.

### 1.2 Description of the intervention for MCI

At present, treatment of MCI mainly includes pharmacologic treatments and nonpharmacologic therapies. Both treatments have been found to ease the cognitive and behavioral symptoms of MCI in some individuals.^[5–7]^ However, Ströhle et al reported that there was no effect of the treatment with cholinesterase inhibitors in patients with MCI (n = 53,693 patients; Row score standardization [SMCR] = 0.03, 95% confidence interval [CI] 0.00–0.005).^[8]^ In addition, the side effects of the drugs are also considered, and nonpharmacologic therapies have been validated and recommended for patients with MCI. They include cognitive-oriented treatments (reality orientation, cognitive behavior therapy); sensory-oriented treatments (music therapy, exercise therapy, aromatherapy, multisensory approaches); emotion-oriented treatments (validation therapy, reminiscence therapy, art therapy, creative story therapy); and behavior-oriented treatments (behavior therapy, bright-light therapy).^[9]^

As one of the nonpharmacologic therapeutic modalities, exercise intervention plays an increasingly important role in prevention, rehabilitation, and health medicine over the past 2 decades. As a protective factor against the occurrence of MCI, exercise intervention improves brain remodeling at many levels including changes in protein and gene expression, in addition to modifications at the subcellular and cellular levels (e.g., rewiring neural pathways and long-term potentiation), that have protective effects on cognitive function in the elderly.^[10]^ Exercise can be done at any time (e.g., playing, travelling, working, engaging in recreational pursuits, and carrying out household chores). Due to various kinds of exercise, there are no special equipment or instruments required, there is a low economic cost, it is readily accessible, etc. Exercise intervention is more suitable for the cognitive management of the MCI population in the community setting.

### 1.3 How does the exercise intervention work for MCI?

Exercise intervention has been shown to have a huge impact on several different aspects of cognitive function, including executive function, processing speed, episodic memory, attention, language, reading, and working memory.^[10]^ However, the effects of the particular exercise intervention methods, the exercise intensity and the duration of exercise intervention on the functioning in various cognitive domains are different. In terms of methods of exercise intervention, combining aerobic exercise with resistance training and flexibility training may improve performance on executive tasks, including working memory, processing speed and attention, more than one form of exercise by itself.^[10]^ In terms of the intensity of the exercise intervention, compared to stretching exercises with a heart rate reserve at or below 50%, exercise with a heart rate reserve of 75% to 85% can significantly improve executive control processes such as cognitive flexibility, multitasking, selective attention, and information processing efficiency.^[11]^ In terms of the duration of exercise interventions, a randomized controlled trial (RCT) showed 6 months of aerobic exercise significantly improved left, right, and total hippocampal volumes, and left hippocampal volume was positively associated with learning performance and verbal memory.^[12]^ Lam reported that weekly engagement in physical activity for 12 months (the exercise conditions included a mind-body exercise (e.g., Tai Chi), an aerobic exercise session (e.g., static bicycle riding) and a stretching and toning exercise) could improve global cognitive ability, delayed recall, and verbal fluency with time (*P* < .05) in people with MCI.^[13]^

### 1.4 Why is it important to do this review?

From the studies presented above, there are conflicting results on the specific cognitive domains affected, with some studies reporting cognitive gains from exercise interventions in memory function or verbal fluency,^[12,13]^ and other studies reporting effects on executive function.^[10,11]^ The changes in the emphasis on or trends in different cognitive domains in the process of developing exercise interventions needs to be further explored. Until now, there has been no published literature on examining a subgroup analysis of the effects of exercise interventions on different cognitive domains in the elderly with MCI. Based on the hypothesis that exercise interventions may have some regular, generalized effects on various cognitive domains of the brain, the aim of this study is to explore the influence of exercise interventions on specific cognitive domains in the elderly with MCI, and produce a preliminary ranking of the degree or magnitude of the influence of certain exercise interventions on various cognitive domains. The study will contribute to revealing the different effects of exercise interventions on improving various cognitive functions.

## 2 Objectives

The aim of this meta-analysis is to explore the effect of exercise interventions on different cognitive domains in old people with MCI. To this end, the proposed study will answer the following questions:

- Do exercise interventions improve cognitive functions in older adults with MCI?
- Do these exercise interventions have different effects on specific cognitive domains?
- Can a subgroup analysis identify the effects of exercise interventions on specific cognitive domains in older people with MCI?

## 3 Methods and analysis

### 3.1 Study inclusion and exclusion criteria

#### 3.1.1 Type of studies

We will include RCTs that used a method of random sequence generation with allocation concealment and are assessed as having low or unclear risk of bias using the Cochrane Collaboration Risk of Bias tool. This strategy helps minimize the inclusion of studies of low quality and high selection bias that are known to inflate effect sizes, and it is a technique that has been used in a number of other systematic reviews and meta-analyses. Quasi-RCTs and crossover trials will not be eligible for inclusion.

#### 3.1.2 Types of participants

Older adults with a diagnosis of MCI were recruited as participants. The diagnostic inclusion and exclusion criteria for the study samples were as follows.

##### 3.1.2.1 Diagnostic criteria

Inclusion criteria: Based on the adaptations of criteria suggested by Petersen that are commonly used to identify “mild cognitive impairment,”^[14,15]^ the participants with MCI should have the following: memory complaint, usually by the patient or family members, and preferably corroborated by an informant; objective cognitive decline that is inconsistent with age and education level; essentially normal general cognitive function as judged by the physician; intact activities of daily living; and absence of dementia. The participants were aged 55 years or older. We will include other measures of cognitive status as determined by the study authors’ own definitions of “mild cognitive impairment,” and record these definitions.

We will analyze the definition of MCI in each study and will contact the author or authors if further clarification is required. If they fail to respond, then the clinical experts of the relevant review team will classify the trials, or we will list them as “studies awaiting classification.”

Exclusion criteria: The participants would be excluded if they meet any of the following: They take medications for cognition, neurologic conditions (e.g., stroke, multiple sclerosis, Parkinson disease), chronic or acute conditions, or depressive symptoms that would preclude exercise. They engage in regular exercise (≥30 min/d, ≥3 d/wk). They have medical histories that include unstable cardiac disease, significant cerebrovascular disease, musculoskeletal impairment, etc. They have other medical conditions with significant psychiatric or metabolic sequelae.

#### 3.1.3 Types of interventions

##### 3.1.3.1 Methods of exercise intervention

The following experimental interventions are eligible: aerobic, resistance, or multicomponent exercise. Additionally, all intervention delivery modes (e.g., personal, group, or dyadic) and support methods (e.g., telephone and internet, face-to-face) are eligible for inclusion. There will be no limit to the professional background of the participants who supported the intervention, and unsupported (self-administered/self-guided) interventions will also be eligible for inclusion.

##### 3.1.3.2 Methods of comparator interventions

The control interventions could be sham, placebo, or no treatment or health education. Examples of appropriate designs are as follows:

- Exercise intervention vs control (no-treatment control; treatment as usual).
- Exercise intervention vs placebo or nonspecific factor component control, such as sham control (e.g., where intervention time is equivalent to that provided in the experimental group but only nonspecific factors are provided as an intervention, such as stretching activities).
- Exercise intervention vs health education.

#### 3.1.4 Types of outcome measures

Studies eligible for inclusion will use scales to measure specific cognitive domain outcome measurements, including general cognition, executive function, memory, language, and visuospatial ability. Where multiple time points are reported, a primary end point ≤6 months posttreatment will be adopted to minimize the likelihood that bias associated with examining only short-term posttreatment effects that are likely to result in higher effect sizes.^[16–18]^

#### 3.1.5 Types of timing

This review will include any kind of exercise interventions. For patients with MCI, a trial lasting for 12 weeks usually shows cognitive enhancement rather than maintenance of cognitive function.^[19]^ Therefore, all trials that reported the results after randomization for at least 12 weeks or longer will be included in our study. There is no minimum duration of follow-up.

#### 3.1.6 Types of intervention settings

There are no restrictions on the location of the intervention. For example, trials where the intervention is delivered in primary care, secondary care, homes, residential care homes, community settings, and university-based clinics will all be included.

### 3.2 Search methods for the identification of studies

#### 3.2.1 Electronic searches

Based on key terms from previous literature reviews and Medical Subject Headings, we will search the following electronic bibliographic databases: MEDLINE, Embase, The Cochrane Library (Cochrane Central Register of Controlled Trials), Web of Science (Science and Social Science Citation Index), China National Knowledge Infrastructure (CNKI), the Wan Fang Database, and CBM (China Biology Medicine). To ensure saturation of the literature, we will further examine the reference list of the included studies and relevant reviews. Meanwhile, we will distribute the bibliography of the included articles to the system review team.

The search strategy will include only terms relating to or describing the intervention. The search strategy we will use for the retrieval of reports of trials from Cochrane is summarized in Table 1. The search strategy will be modified as necessary for other databases.

**Table 1:**
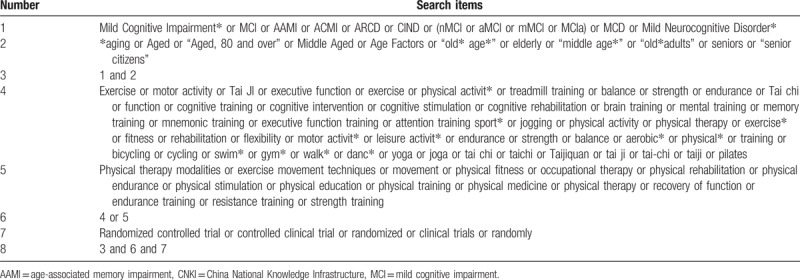
Search strategy for Cochrane Library.

There will be no language restrictions. Studies published between January 1990 and the date the searches are run will be sought. The searches will be re-run just before the final analyses and additional studies retrieved for inclusion.

#### 3.2.2 Searching other resources

Relevant RCTs and reviews for additional studies will be hand searched for further eligible studies. On the one hand, journals containing the highest numbers of included studies will be hand searched for recent potentially eligible publications (≤12 months); On the other hand, we will contact experts in the field of MCI and dementia to identify any unpublished or ongoing trials. In addition, the WHO International Clinical Trials Registry Platform (ICTRP) and ClinicalTrials.gov will be checked to identify planned, ongoing, or unpublished trials.

### 3.3 Data collection and analysis

#### 3.3.1 Selection of studies

**Figure 1:**
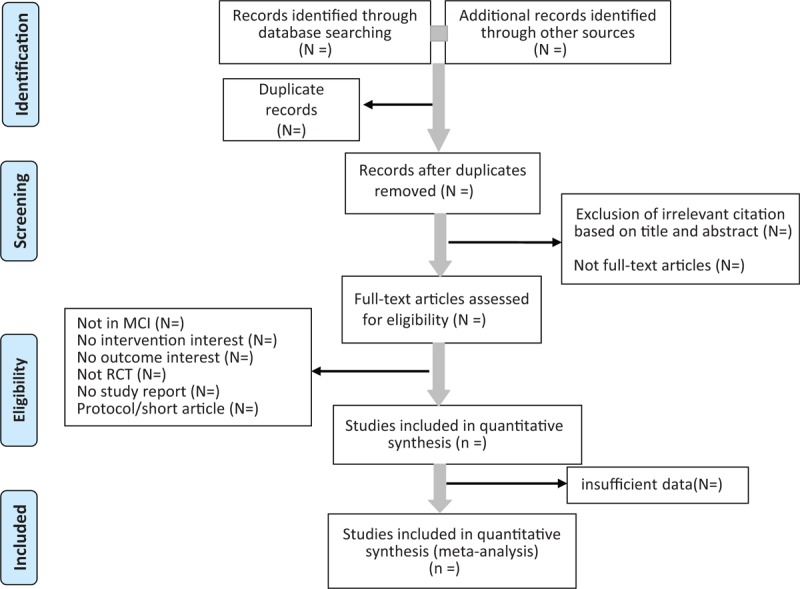
Flow diagram for study selection process used in this meta-analysis.

Two researchers will act as reviewers and independently screen titles and abstracts of studies. Duplicates will be omitted using EndNote software (V.X7.8).^[20]^ Relevant studies will be selected according to the predefined inclusion criteria. If necessary, reviewers will examine full-text reports to identify eligible studies. EndNote software will also be used to manage records. If consensus cannot be attained, a 3rd member of the study team will be contacted. Any disagreement will be resolved by consensus. An Excel spreadsheet has been developed to manage all review data. We will illustrate the selection process in a PRISMA diagram (Fig. 1).^[21]^

#### 3.3.2 Data extraction and management

Search results will be uploaded into an EndNote database (X.7.8) and de-duplicated. Two review authors, working independently, will extract trial information using a standardized and piloted extraction method, referring also to a guidance document, and resolving discrepancies by discussion, or by the involvement of a 3rd review author.

Data will be double extracted by the two reviewers using a data extraction form developed in Excel for this review. Where possible, we will extract the following information related to characteristics of participants, the intervention, and the study design, and contact with the study authors in the event of missing data and any uncertainties. The detailed information will be extracted using a predetermined data form and includes the following (Table 2): general information, details of study, design, study subjects, intervention characteristics, outcome.

**Table 2:**
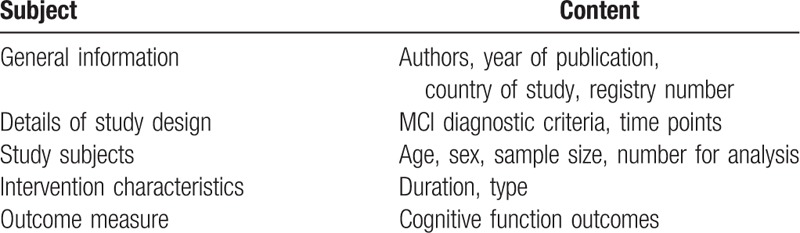
Data and information extraction schedule.

#### 3.3.3 Risk of bias of assessment

*Cochrane Handbook for Systematic Reviews of Interventions* will be used to assess the risk of bias.^[22]^ The following risk of bias domains will be assessed: sequence generation, concealment of allocation, blind subjects and therapists, blinded outcome assessment, selective outcome reporting, incomplete outcome data, and other bias. According to PEDpro, another 3 items will be added, including “the type of statistical analyses used (true intention-to-treat vs other),” “eligibility criteria,” and “baseline comparability.”^[23]^ Details of the qualitative assessment are shown in Table 3.

**Table 3:**
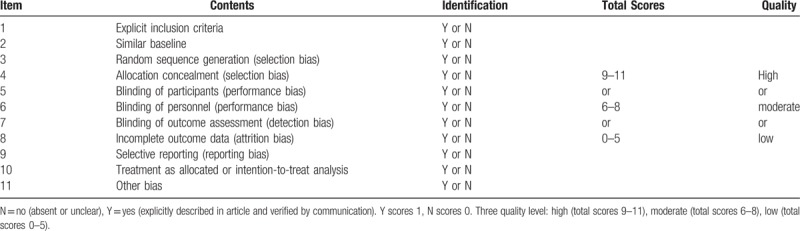
Modified assessment form.

Study quality ratings will be compared and the 3rd reviewer will be contacted if consensus is not attained. If all domains are at low risk of bias, the overall risk of bias of individual studies will be categorized as low risk of bias. Otherwise, overall risk of bias will be categorized as high risk of bias. The “risks of biases” summary will be presented graphically.

#### 3.3.4 Data synthesis and statistical analysis

Continuous outcomes will be analyzed using weighted mean differences (with 95% CI) or standardized mean differences (95% CI) if different measurement scales are used.^[24]^ All analyses will be conducted with Review Manager V.5.3 software. If a meta-analysis is not possible, we will provide a narrative summary of the results from individual studies. The choice of fixed or random effects models will be guided by the level of statistical heterogeneity.

#### 3.3.5 Dealing with missing data

Missing data in the individual trials may put the study estimates of effects at a high risk of bias.^[22]^ We will attempt to contact the original authors of the study to obtain the relevant missing data. Important numerical data will be carefully evaluated. If missing data cannot be obtained, an imputation method will be used. The intention-to-treat principle will be followed as far as possible, analyzing all patients as they were randomized.

#### 3.3.6 Assessment of heterogeneity

The presence of statistically significant heterogeneity will be examined by calculating the *Q* statistic with the quantification of the degree of heterogeneity calculated using the *I*^2^ statistic. If high levels of heterogeneity among the trials exist (*I*^2^ ≥ 50% or *P* < .1), statistically significant heterogeneity is indicated.^[25,26]^ We will try to explain the source of heterogeneity by the subgroup analysis.

Subgroup analysis and investigation of heterogeneity:

- A subgroup analysis for specific cognitive domains will play an important role in ascertaining the most sensitive cognitive domain that has noticeable improvements by exercise intervention. Impairments in 5 specific cognitive domains will be analyzed, including general cognition, executive functions, memory, language, and visuospatial ability.
- Subgroup analyses will be used to explore possible sources of heterogeneity. We will test the heterogeneity by considering the variability in participant factors (e.g., age, sex) and trial factors (baseline severity of cognitive impairment, follow-up period, intervention type, type of control group).

#### 3.3.7 Sensitivity analysis and publication bias

Sensitivity analysis will be conducted by individually omitting each study from the meta-analysis to examine whether the effect size was biased by the inclusion of any particular study. Selective outcome reporting bias^[27]^ will be examined using STATA 12.0 to detect the publication bias.^[28]^

## 4 Discussion

Along with the world's population ages, MCI has become a major aging problem, with continuously increasing prevalence throughout the world. Currently there is no cure for MCI, but the identification and targeting of modifiable risk factors such as exercise may offer the opportunities to modify its onset and course. Some aerobic exercise, resistive exercise and mind–body exercises, such as walking, running, mechanical resistance training, Tai Chi, Qigong, and Yoga, are often recommended to promote cognitive function for patients with MCI. A meta-analysis demonstrated that aerobic exercise beneficial effects on attention, delayed recall, and reaction time.^[29]^ Another meta-analysis showed that exercise invention may improve executive function.^[30,31]^ A previous Cochrane review that included 12 RCTs of aerobic exercise for older people showed there was no beneficial effect on cognitive impairment.^[32]^ To the best of our knowledge, there is no studies could provide strong evidence about examining a subgroup analysis of the effects of exercise interventions on different cognitive domains in the elderly with MCI. We hope to provide more practical and targeted results investigating the effect of exercise invention for cognitive function in the current systematic review and meta-analysis.

We make a modified assessment form which incorporates the advantages of Cochrane assessment tool and PEDro scale, making our qualitative evaluation more reasonable and credible.

Although we took account into methodology carefully, it should be noted that there might be several limitations have to be mentioned regarding our systematic review and meta-analysis. One limitation of this review is that the reliability of the results will largely depend on the comprehensiveness and the methodologic quality of the primary studies included in this review. Another limitation is that different area of participants and types of exercise inventions may run risk of heterogeneity.

### 4.1 Ethics and dissemination

Ethical approval is not required for the present systematic review. The protocol was registered with the International Prospective Register of Systematic Reviews (PROSPERO) on June 5, 2018 (registration number: CRD42018093902). Any future amendments will be documented on the PROSPERO website.

## Author contributions

Lina Wang, Xianglian Zhou, Xinhua Shen conceived the study. The protocol was drafted by Xianglian Zhou and Lina Wang, and revised by Lina Wang and Xinhua Shen. Both Xianglian Zhou and Lina Wang serve as the first author. Xianglian Zhou and Xia Zhao developed the search strategy. Xianglian Zhou and Jie Wang will independently work on study selection, quality assessment, data extraction, and synthesis. All authors have read and approved the final version of the manuscript.

**Conceptualization:** Xianglian Zhou, Lina Wang, Xin-Hua Shen.

**Data curation:** Jie Wang, Xia Zhao.

**Formal analysis:** Xianglian Zhou.

**Methodology:** Xianglian Zhou, Lina Wang, Jie Wang, Xinhua Shen.

**Software:** Xianglian Zhou, Lina Wang.

**Supervision:** Xin-Hua Shen.

**Writing – original draft:** Xianglian Zhou, Lina Wang.

**Writing – review & editing:** Xianglian Zhou, Lina Wang.
